# Application of Lipophilic Prodrug Charge Masking Strategy to Obtain Novel, Potential Oxytocin Prodrugs

**DOI:** 10.3390/ijms26104772

**Published:** 2025-05-16

**Authors:** Agata Gitlin-Domagalska, Anna Olejnik, Jarosław Ruczyński, Dominika Starego, Natalia Ptaszyńska, Anna Łęgowska, Dawid Dębowski, Chaim Gilon, Krzysztof Rolka

**Affiliations:** 1 Department of Molecular Biochemistry, Faculty of Chemistry, University of Gdańsk, Wita Stwosza 63, 80-308 Gdańsk, Poland; agata.domagalska@ug.edu.pl (A.G.-D.); dominika.starego@phdstud.ug.edu.pl (D.S.); natalia.ptaszynska@ug.edu.pl (N.P.); anna.legowska@ug.edu.pl (A.Ł.); dawid.debowski@ug.edu.pl (D.D.); krzysztof.rolka@ug.edu.pl (K.R.); 2 Department of Biotechnology and Food Microbiology, Faculty of Food Science and Nutrition, Poznań University of Life Sciences, Wojska Polskiego 48, 60-627 Poznań, Poland; anna.olejnik@up.poznan.pl; 3 Institute of Chemistry, The Hebrew University of Jerusalem, Jerusalem 91904, Israel; chaimgilon@gmail.com

**Keywords:** oxytocin, prodrugs, permeability, PAMPA, Caco-2

## Abstract

A Lipophilic Prodrug Charge Masking (LPCM) strategy involves masking of hydrophilic peptide charges with alkoxycarbonyl groups, which are cleaved by esterases after intestinal absorption. This study investigates the LPCM strategy’s applicability to oxytocin (OT), a peptide with well-defined biological activity. A series of OT prodrugs with varying alkoxycarbonyl chain lengths (2 to 12 carbon atoms) were synthesized, and their permeability was assessed using parallel artificial membrane permeability assay (PAMPA) and Caco-2 cell culture models. The PAMPA results indicated that OT demonstrated poor permeability (P_app_ = 2.2 × 10^−6^ cm/s), while its prodrugs Hoc-OT, Oct-OT, and Dec-OT were characterized by significantly better permeability, with Dec-OT achieving a four-fold increase over OT. The prodrug with a 12-carbon chain (Dod-OT) exhibited poor permeability; however, its high mass retention suggests strong membrane affinity. Further evaluation, using the Caco-2 cell model, revealed a 1.8-fold higher P_app_ of Oct-OT compared to OT, indicating possible higher oral availability. Conversely, Hoc-OT exhibited lower permeability than OT. Our findings indicate that the LPCM strategy can effectively boost the oral bioavailability of certain peptides, paving the way for their transformation into bioavailable drugs.

## 1. Introduction

Understanding and clarifying the physiological function and structures of many biologically relevant peptides, such as hormones, neurotransmitters, and growth factors has an enormous impact on current pharmaceutical advancements. Major development of peptide drugs started with isolation in 1921 and commercial production in 1923 of insulin [1], described as a “miracle drug”. Peptides are highly selective, remarkably efficient, and their metabolites are not toxic. However, their application as drugs is restricted due to certain drawbacks such as susceptibility to proteolytic degradation, which is important for peptides activation or removal of these that are no longer needed but seriously limits their application due to low stability in the intestine and plasma [2]. Moreover, most biologically active peptides are usually characterized by high hydrophilicity, which enables their solubility in water, but on the other hand, it lowers their permeability through the body membranes resulting in low bioavailability [2,3].

Current medicinal chemistry focuses on improving peptides’ proteolytic stability and bioavailability without reducing their activity. This is a particularly challenging task, and it requires careful and well-considered design of specific modifications of peptides, acknowledging various aspects such as absorption and transportation from the administration spot to the specific place of action [4,5]. The improvement in peptides resistance to the proteolysis was well mastered; the most common strategies include cyclization of peptide backbone, introduction of non-proteinogenic amino acids (e.g., D-enantiomers), N-methylation of positions susceptible to proteolysis [4,5,6].

Oxytocin (OT) is a nonapeptide neurohormone produced in the hypothalamus, well-characterized for its involvement in labor and lactation but also social behavior, as well as affection. Its improper level was found in people suffering from Autism Spectrum Disorders (ASD) [7,8,9]. ASD is a group of neurodevelopmental symptoms with a specific pattern of abnormalities in social manners, manifested by problems with expressing emotions, which further lead to difficulties in communication and dealing with different social situations [10]. Existing treatments focus mainly on behavioral therapy, and there is no specific and effective drug treatment for the core dysfunctions of ASD reported. Those that are currently in use treat some symptoms like irritability and repetitive behavior, but they cause many side effects, and patients with autism may respond atypically [11].

Treatment with OT may improve social abilities and emotions recognition in autistic individuals [10,12]. Thus, the suggestion to deliver it directly to cerebrospinal fluid emerged. The intranasal route of drug administration, which allows for omitting the blood–brain barrier and delivering therapeutics directly to the central nervous system, seemed to answer these needs. Some studies show that daily administration of intranasal OT contributes to the improvement of ASD symptoms and increases social abilities, while the other group showed no efficacy in administering OT intranasally to autistic adolescents [10,12]. These differences are not necessarily caused by therapeutic inefficiencies of OT but the route of administration and imperfectness of used devices, resulting in dose and efficacy diversity [13]. In addition, ASD children with communication problems and social dysfunctions have significant difficulty tolerating intranasal sprays. Oral administration of drugs is considered to be most convenient not only for ASD but also for pediatric and geriatric patients [14,15]. Interestingly, Zhuang et al. [16] indicated that OT administered orally can alter neural responses and has therapeutic potential in ASD, similarly to intranasal application; however, it may offer better tolerance, especially in long-term treatment. All of the above underline that in some cases, oral administration seems the most convenient and is crucial for conserving the good effectiveness of therapy. Therefore, while intranasal administration of OT shows promise, it is not without its challenges and limitations, particularly in terms of device efficacy and patient tolerance.

OT is only one of many examples of peptide drugs that preferably should be administered orally. Thus, the oral delivery of therapeutic peptides and proteins remains a significant challenge in drug development due to their susceptibility to degradation and poor intestinal permeability [17]. OT has gained interest as a potential therapeutic target for various conditions. However, the poor oral bioavailability of OT has hindered its clinical application [18,19]. While OT’s bioavailability is crucial for its therapeutic applications, challenges remain in achieving consistent and effective delivery, particularly through non-invasive routes. Research indicates significant variability in OT’s bioavailability depending on the route of administration and individual factors. According to Groot et al. [20], after sublingual administration, OT exhibits poor bioavailability ranging from 0.007% to 0.07%, with a lag time of 0.12 to 0.30 h. Yamasue et al. [21] reports novel nasal OT spray (TTA-121) with enhanced (3.6 times higher) bioavailability when compared to standard spray allowing effective dosing in clinical trials. What limits OT’s bioavailability is also its short plasma half-life, typically between 1 and 6 min, and it is rapidly cleared by the liver and kidneys [22]. In the case of intranasal administration, peak levels are reached within 15–30 min, followed by a quick decline [23]. Summing up, variability in the bioavailability of certain peptides can be attributed to individual physiological differences, the administration method and the formulation of the drug [24].

Recent research has explored the use of prodrugs as means to enhance the peptides’ intestinal permeability and improve their oral absorption [18,25]. Prodrug strategies include the chemical modification (removed before binding to the molecular target) of a drug molecule to improve its physicochemical properties and enhance its absorption. They have been employed to modify the physicochemical properties of bioactive peptides, such as solubility and lipophilicity, in order to facilitate their transport across the intestinal epithelium [4,6,18]. Additionally, studies have investigated the potential of OT prodrugs to modulate tight junction permeability, thereby enhancing the paracellular transport of the drug [17,26]. One of such prodrug approaches is Lipophilic Prodrug Charge Masking (LPCM) strategy, first described in 2018 by Schumacher et al. [27]. The core idea of LPCM is the transitional masking of hydrophilic peptides’ charge by alkoxycarbonyl groups. These modifications, connected by a cleavable ester bond, are supposed to be removed by esterases after permeating through the intestine to the bloodstream. Cyclic N-methylated hexapeptides containing an Arg-Gly-Asp (RGD) integrin-binding motif were chosen as representatives of active, hydrophilic, charged peptides for initial studies [18]. Analogs with masked Arg and Asp residues were synthesized. Two hexyloxycarbonyl (Hoc) groups were linked to the guanidinium group of Arg, and the β-carboxyl group of Asp was converted into methyl ester (OMe). The permeability of parent peptides and corresponding prodrugs was tested in vitro using the intestinal Caco-2 model. All of the studied parent peptides were characterized by low permeability in vitro. Their P_app_ (Permeability Coefficient) values were much lower (even 20-fold in the apical to basolateral direction) than those of their modified analogs. Moreover, the prodrug 12P (nomenclature according to [27]) selected for more detailed analyses was stable in brush border membrane vesicles (BBMVs), but it was quickly degraded in plasma (within 5 min) to give parent peptide 12. Pharmacokinetic studies performed in rats showed that the LPCM approach improved the bioavailability of the prodrug 12P over 70-fold compared to peptide 12. Interestingly, this strategy converted absorption mechanism of the polar peptides from paracellular to transcellular, which significantly affects the oral availability of peptides. This excellent outcome encouraged us to look closer into LPCM as a way to transform peptides into bioavailable drugs.

Results presented by Schumacher et al. [27] give strong foundations for further study of LPCM. It is important to indicate that in the original paper only short, cyclic, model peptides, characterized by high stability, designed specifically for this research, were investigated, and LPCM was not applied to biologically active peptides. In our work, for the first time, we verify LPCM’s strategy applicability to a certain peptide with well-defined and described biological activity. OT was chosen as a model peptide, due to its well-described important biological function and therapeutic potential limited by poor oral bioavailability and relatively easy chemical synthesis. We synthesized a series of its prodrugs where the N-terminal amino group was masked by various alkoxycarbonyl groups differing in alkyl chain length, from 2 to 12 carbon atoms, as summarized in Table 1. We examined their permeability using parallel artificial membrane permeability assay (PAMPA) and Caco-2 cell culture to verify the LPCM hypothesis applicability in case of biologically active peptides.

## 2. Results

### 2.1. Peptide Synthesis

In order to examine LPCM applicability to produce OT prodrugs, a series of OT analogs was synthesized. In short, the N-terminal amino group of OT was masked by esterase-cleavable alkoxycarbonyl groups differing in alkyl chain length to test how it influences peptide’s solubility and permeability. We obtained eight OT analogs with alkoxycarbonyl group containing 2 (ethyloxycarbonyl-), 3 (propyloxycarbonyl- and 2-methoxyethyloxycarbonyl), 4 (butyloxycarbonyl-), 6 (hexyloxycarbonyl-), 8 (octyloxycarbonyl-), 10 (decyloxycarbonyl-), and 12 (dodecyloxycarbonyl-) carbon atoms in an alkyl chain.

### 2.2. PAMPA

The permeability of OT and its prodrugs was estimated by the PAMPA test (Corning, Gentest PAMPA Plate System). The PAMPA method is a common method for the permeability evaluation [26,28,29,30] of early drug candidates. Although this system lacks transporters, paracellular pathway enzymes, and other cell-associated processes, it measures passive diffusion, the primary mechanism by which drugs traverse biological barriers. Consequently, due to its low cost, high throughput, and relative handling ease, it serves as screening tool for predicting drugs absorption potential [31,32]. The PAMPA reflects the passive transport through biological membranes, and its results are reported to correlate with those obtained from the Caco-2 cell model [33,34,35].

The results of the PAMPA were expressed as permeability coefficient P_app_ and compared to the low and good permeability markers, i.e., atenolol and caffeine, respectively (Table 2). According to the plates provider, the P_app_ higher than 4.0 × 10^−6^ cm/s characterizes compounds with good permeability, while compounds with P_app_ below this value are considered to have low permeability.

Oxytocin demonstrated poor permeability (P_app_ = 2.2 × 10^−6^ cm/s), which was the expected result due to its hydrophilic characteristic. Permeability was improved for its analogs. However, prodrugs bearing alkoxycarbonyl groups of up to three carbon atoms were characterized by relatively low permeability (P_app_ < 3.0 × 10^−6^ cm/s). Prodrugs with the N-terminal group masked by hexyloxycarbonyl, oxtyloxycarbonyl, and decyloxycarbonyl groups (i.e., 6, 8, and 10 carbon atoms, respectively) showed better permeability, which was improved over four-fold in case of Dec-OT when compared to unmodified OT. Interestingly, a growing alkyl chain length did not correlate with better transport. Dod-OT, a prodrug with a 12-carbon atoms masking group, demonstrated poor P_app_ = 1.08 × 10^−6^ cm/s, which is less than that for unmodified OT. However, Dod-OT is characterized by high mass retention (67%), which means that part of the compound remained in the artificial membrane, suggesting it has a strong affinity for the membrane [35,36]. High mass retention, at least 30%, was observed also for Prop-OT and But-OT.

The PAMPA was performed for the initial screening of the passive transport of OT and its prodrugs, and compounds demonstrating the best permeability were selected for further research using the Caco-2 cell model. These includes Hoc-OT, Oct-OT and Dec-OT. We also included OT as a reference. Dod-OT was selected for the cell assay despite its low P_app_ value, as its high mass retention may cause the underestimated permeability in the PAMPA.

### 2.3. Permeability Experiments in the Caco-2 Model

The human colon carcinoma cell line Caco-2 is the most commonly used cell-based system to predict drug intestinal permeability. Caco-2 cells spontaneously differentiate into enterocyte-like cells and form a monolayer characterized by the tight junctions between the cells with the apical brush border, typical of the small intestine epithelium [37]. The differentiated Caco-2 cell monolayer expresses many brush border enzymes on the apical membrane, including small intestinal hydrolases, some cytochrome P-450 isoenzymes, and phase II enzymes such as glutathione-*S*-transferases, sulfotransferase, and glucuronidase. Moreover, many active transport systems found in enterocytes have been described in Caco-2 cells. Thus, the differentiated Caco-2 cell culture model exhibits enterocytes’ morphological and functional characteristics and is considered the model of a small intestinal epithelial barrier [38,39,40]. The Food and Drug Administration and European Medicines Agency have approved the Caco-2 cell line as a reliable in vitro model for predicting human oral drug bioavailability [41,42]. A good correlation between permeability in the Caco-2 model and oral drug absorption in humans was based on previous studies in this cell line [43].

The Caco-2 cell monolayer development and tight junction formation during the long-term cell culture on the semipermeable membrane in a two-compartment system were monitored by measuring the transepithelial electrical resistance (TEER). The Caco-2 intestinal epithelium models characterized by high TEER values of at least 600 Ohm/cm^2^ were applied in permeability experiments (Figure S19).

**Figure 1:**
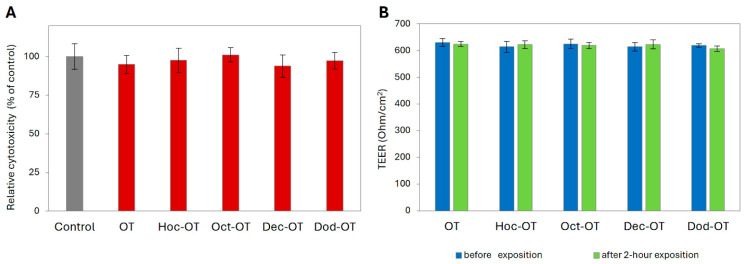
Effect of oxytocin (OT) and its derivatives (Hoc-OT, Oct-OT, Dec-OT, Dod-OT) at a concentration of 100 µg/mL on Caco-2 cell viability (**A**) and Caco-2 monolayer integrity (**B**). Caco-2 monolayer integrity was evaluated by transepithelial electrical resistance (TEER) assessment before and after 2 h treatment with the analyzed compounds. Data represent mean values ± SD (*n* = 3).

Before permeability experiments, the cytotoxicity and the effect of OT and its derivatives (Oct-OT, Hoc-OT, Dec-OT, Dod-OT) on Caco-2 intestinal barrier integrity were examined. This assessment was performed using a dose (100 µg/mL) and treatment time (2 h) similar to those applied in the intestinal transport analysis. No cytotoxicity of the tested compounds at a concentration of 100 µg/mL after a 2 h exposure was observed (Figure 1A). Moreover, the TEER values measured in Caco-2 cell models before and after treatment with OT and its analogs indicated stable and high monolayer integrity (Figure 1B).

**Figure 2:**
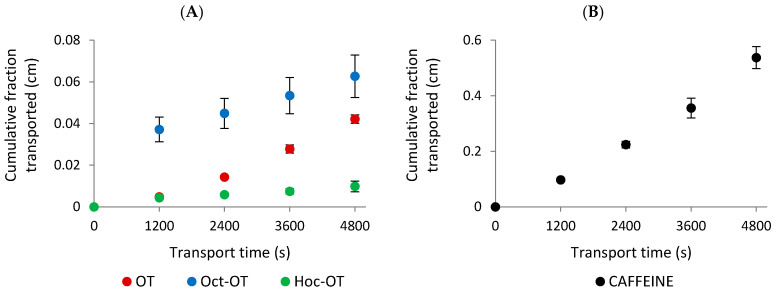
The experimentally determined “cumulative fraction transported” of oxytocin (OT) and its derivatives (Oct-OT and Hoc-OT) (**A**) and caffeine (**B**) in the apical to basolateral transport across the Caco-2 cell monolayer. Data represent mean values ± SD (*n* = 3).

The transport of OT and its derivatives (Oct-OT, Hoc-OT, Dec-OT, Dod-OT) selected for further in vitro studies was evaluated at a target concentration of approximately 100 µg/mL. Intestinal transport was initiated by adding the tested compounds to the apical compartment for absorptive transport determination. The solutions from the acceptor compartments were sampled at 20-minute intervals to analyze the concentration of transported compounds. The transport experiments showed that OT, Oct-OT, and Hoc-OT penetrated the Caco-2 intestinal epithelial barrier (Figure 2A). In contrast, Dec-OT and Dod-OT were undetectable in the fractions transported from the apical to the basolateral (acceptor) compartment, which prevented the prediction of their intestinal permeability in the Caco-2 model. This probably results from high hydrophobicity of compounds and, thus, high affinity and accumulation in the cell membrane.

**Figure 3:**
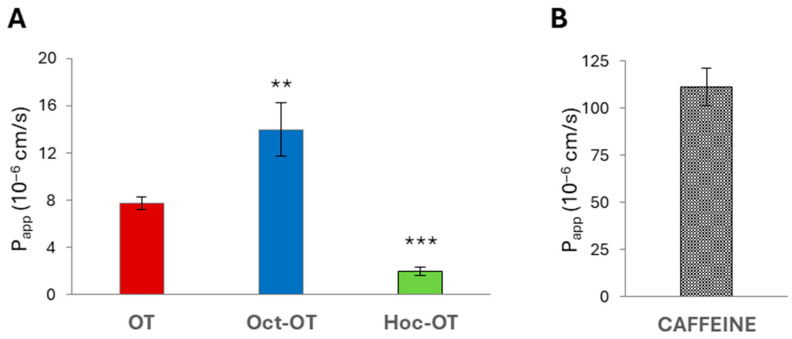
The apparent permeability coefficient (P_app_) of oxytocin (OT) and its derivatives (Oct-OT and Hoc-OT) (**A**) and caffeine (**B**) determined in the Caco-2 intestinal epithelium model. Data represent mean values ± SD (*n* = 3). The statistical analysis assessed differences between P_app_ calculated for OT and its analogs with significance of ** *p* ≤ 0.01 and *** *p* ≤ 0.001.

The results indicated significant differences in the absorptive intestinal transport of OT and Oct-OT and Hoc-OT. The cumulative fraction transported (CFT) in the receiver compartment increased linearly with time (Figure 2A). The time-course experiments showed that the CFT of Oct-OT was significantly higher than the CFT of OT. In contrast, Hoc-OT was characterized by a lower CFT than OT (Figure 2A). It was reflected in the apparent permeability coefficient (P_app_) values determined for the compounds analyzed. The P_app_ value calculated for Oct-OT (14.00 ± 2.27 × 10^−6^ cm/s) was approximately 1.8-fold higher than that obtained for OT (7.74 ± 0.53 × 10^−6^ cm/s), suggesting relatively high Oct-OT oral availability. However, comparatively poor intestinal bioavailability of Hoc-OT was noted, as indicated by a significantly lower P_app_ value (1.99 ± 0.33 × 10^−6^ cm/s) in the Caco-2 cell assay (Figure 3A).

In this study, the caffeine permeability test was performed to confirm the suitability of the Caco-2 cell model to determine transepithelial transport. Caffeine is recognized as a reference substance for which high permeability has been documented [44,45]. The results obtained from transport studies confirmed the high intestinal caffeine permeability with P_app_ value 111.10 ± 9.89 × 10^−6^ cm/s (Figure 2B and Figure 3B), which proves Caco-2 monolayer functionality and its suitability to evaluate the intestinal transepithelial transport of OT derivatives.

## 3. Discussion

Alkyl chain elongation, which aims at improvement of lipophilicity, does not always correlate with permeability improvement, which is reflected in both PAMPA and Caco-2 results. Interestingly, PAMPA and Caco-2 assay outcomes do not correlate with each other, and even though Hoc-OT revealed improved permeability when compared to OT in the PAMPA, the results of Caco-2 were the opposite. The reason for this may be the presence of proteases in the Caco-2 model, which may disturb the results by degrading peptides, thereby decreasing the real amount of permeated drug/prodrug [38,39]. The PAMPA, which is based on an artificial membrane, considers only passive transport mechanisms, such as transcellular passive diffusion, and does not account for active transport processes, including active influx and efflux transporters, which may significantly affect the ability of compounds to cross the barrier. The lipid composition, pH, and other conditions in the PAMPA can be adjusted to mimic different biological barriers, but they still do not fully replicate the dynamic environment of living cells found in Caco-2. PAMPA is useful for high-throughput screening of passive permeability; however, it may not always predict the complex interactions that occur in the human intestine as accurately as Caco-2 assays. Additionally, multidrug resistance proteins, such as P-glycoprotein, are expressed in the Caco-2 model and can reduce the bioavailability of drugs by actively pumping them back into the intestinal lumen. These active transport processes are not accounted for in the PAMPA, highlighting the need for complementary in vitro models to fully understand the permeability and bioavailability of compounds [46,47].

Compounds with higher hydrophobicity (attached longer alkyl chain) exhibited higher mass retention. High mass retention and low permeability of these compounds may be partly explained by their high hydrophobicity (long alkyl chains are likely to stick to the membrane), which likely favors strong hydrophobic interactions with the membrane, leading to their adsorption on the membrane surface and preventing them from crossing the membrane barrier [48,49,50]. These findings suggest the existence of a tiny balance between the hydrophobicity of peptides and the ability to cross the gastrointestinal barrier, which is so important for oral drug absorption. Thus, when designing new peptide prodrugs used in the LPCM strategy, the hydrophobicity (carbon chain length) of the attached alkyl chain should be carefully considered.

Our study proved that LPCM may be a promising approach to produce novel prodrugs of peptide drugs with increased permeability; however, it needs further investigation and development.

## 4. Materials and Methods

### 4.1. Peptide Synthesis

OT was synthesized using Fmoc chemistry on a Prelude Peptide Synthesizer (Protein Technologies, Inc., Tucson, AZ, USA). Each peptide was synthesized on Rink Amide resin (loading 0.646 mmol/g, GL Biochem, Shanghai, China) as described in our previous papers [51,52]. The protected derivatives of all fluorenylmethoxycarbonyl-amino acids (Fmoc-aa) were purchased from various commercial sources. The peptide chain was elongated in the consecutive cycles of deprotection and coupling. Deprotection was performed with 20% piperidine in dimethylformamide (DMF), and peptide chains elongation was performed using 3-fold molar excess of each Fmoc-protected amino acid and 2-(1*H*-Benzotriazole-1-yl)/ 1-hydroxybenzotriazole/*N*-Methylmorpholine (Fmoc-aa/TBTU/HOBt/NMM) (molar ratio 1:1:1:2). Masking groups were introduced using corresponding alkyl chloroformates on resin after *N*-terminal cysteine Fmoc deprotection. In short, alkyl chloroformate and diisopropylethylamine (DIPEA) (molar ratio 1:2) in DMF were added to the peptidyl resin, and the reaction time was 1–20 h. Coupling was repeated until a negative chloranil test. After completing the synthesis, peptide was cleaved from the resin, and the protecting groups were removed in a one-step procedure using a mixture of trifluoroacetic acid (TFA)/phenol/triisopropylsilane/H_2_O (88:5:2:5, *v*/*v*/*v*/*v*). Subsequently, the disulfide bridge was formed using a 0.1 M methanolic iodine solution.

Obtained crude compounds were purified by reverse-phase high performance liquid chromatography (RP-HPLC) on a PLC 2050 Gilson HPLC with Gilson Glider Prep. Software (Gilson, Villiers-le-Bel, France), equipped with a Grace Vydac C18 (218TP) HPLC column (22 × 250 mm, 10 μm, 300 Å, Resolution Systems). The solvent systems were 0.1% TFA (A) and 80% acetonitrile in A (B). The purity of each peptide was checked by RP-HPLC on a Shimadzu Prominence-I LC-2050C 3D equipped with a Dr. Maisch ReproSil-Pur Basic-C18 (150 × 4.6 mm, 5 μm) and a UV-Vis detector. A linear gradient from 10% to 90% B for 20 min, flow rate 1 mL min^−1^, and detection at λ = 214 nm was used. The mass spectrometry analysis of the synthesized compounds was carried out on MALDI MS (Autoflex maX MALDI-TOF spectrometer, Bruker Daltonics, Bremen, Germany) using an α-cyano-4-hydroxycinnamic acid and/or 2,5-dihydroxybenzoic acid matrix. Mass spectra and HPLC analyses of synthesized compounds are given in the Supplementary Information. ClogP was calculated using ChemDraw Professional 15.0.0.106 software (PerkinElmer Informatics, Inc., Waltham, USA).

### 4.2. PAMPA

A Gentest Pre-coated PAMPA Plate System was purchased from Corning^®^ (Tewksbury, MA, USA). The assay was performed according to the provider instructions, following Caco-2 convention, where the top well is a donor well. The 10 µM solutions of OT, prodrugs, and permeability markers were prepared in PBS buffer, pH 7.4, from 1 mM stock solutions in dimethyl sulfoxide (DMSO). A total of 300 µL per well of tested compounds was added to the donor, filter plate, which, after distribution of all samples, was coupled with acceptor plate filed with 200 µL of phosphate-buffered saline (PBS) per well. All compounds were measured in triplicate. After 5 h of incubation at 25 °C, 200 µL samples were collected from acceptor and donor wells and analyzed using UHPLC-MS to determine concentrations of compounds. The P_app_ and mass retention R were calculated according to following formulas:

$$P_{app}=\frac{-ln[1-\frac{C_{A}}{C_{equilibrium}}]}{A\times (1/V_{D}+1/V_{A})\times t}$$

$$R=1-\frac{C_{D}\times V_{D}+C_{A}\times V_{A}}{C_{0}+V_{A}}$$

where C_0_ = initial compound concentration in donor well, C_D_ = compound concentration in donor well after incubation, C_A_ = compound concentration in acceptor well after incubation, V_D_ = donor well volume (0.3 mL), V_A_ = acceptor well volume (0.2 mL), C_equilibrium_ = [C_D_ × V_D_ + C_A_ × V_A_]/(V_D_ + V_A_), A = filter area (0.3 cm^2^), and t = incubation time in s.

### 4.3. Caco-2 Cell Model Assays

#### 4.3.1. Caco-2 Intestine Epithelial Model

The Caco-2 cell line (ATCC HTB-37), derived from human colon adenocarcinoma, was obtained from the American Type Culture Collection (ATCC, Manassas, VA, USA). Caco-2 cells were cultured at 37 °C under a 5% CO_2_ atmosphere using Dulbecco’s Modified Eagle’s Medium (DMEM), supplemented with 20% (*v*/*v*) fetal bovine serum, non-essential amino acids 100×, and gentamycin (50 mg/mL). For experiments, the Caco-2 cells were plated at a density of 4 × 10^5^ cells per cm^2^ on polyethylene terephthalate (PET) capillary pore membranes with a pore size of 0.4 μm and a growth surface area of 1.13 cm^2^ (Greiner PET hanging). The culture medium was replaced every 2 days. All experiments were performed on differentiated Caco-2 cell monolayers 21 days post-seeding. To check the integrity of the Caco-2 monolayer, transepithelial electrical resistance (TEER) was measured by employing the Millicell Electrical Resistance System (Millipore, Bedford, MA, USA). Moreover, fluorescein-5(6)-sulfonic acid was used as a transport marker, added to the apical side of the Caco-2 cultures at 200 μg/mL concentration. Fluoresceinated sulfonic acid, with a molecular weight of 478 Da, is a hydrophilic, charged molecule at physiological pH and is considered cell impermeable. Unless specified otherwise, all chemicals were purchased from Merck KGaA (Darmstadt, Germany).

#### 4.3.2. Cytotoxicity Test

The effect of analyzed compounds on Caco-2 cell viability was examined at a dose (100 μg/mL) and treatment time (2 h) similar to those applied in the permeability experiment. Cytotoxicity was determined by the lactate dehydrogenase (LDH) test. This method measures the activity of cytoplasmic LDH, which is rapidly released from damaged cells into the cell culture medium when the plasma membrane is damaged due to cell death. The released LDH was determined using the CytoTox-One™ Homogeneous Membrane Integrity Assay, according to the manufacturer’s protocol (Promega GmbH, Mannheim, Germany). LDH released into the culture medium was measured with a coupled enzymatic assay, in which lactate, NAD^+^, and resazurin were used as substrates in the presence of diaphorase. As a result, the conversion of resazurin into a fluorescent resorufin product was noted proportionally to the amount of LDH. Fluorescence was measured at excitation and emission wavelengths of 560 nm and 590 nm, respectively, using a Tecan M200 Infinite microplate reader (Tecan Group Ltd., Männedorf, Switzerland).

#### 4.3.3. Transepithelial Transport Experiment

The analyzed compounds at non-cytotoxic concentration of approximately 100 μg/mL were prepared in Hank’s balanced salt solution (HBSS), which was adapted as a transport medium. Moreover, transport of caffeine as a reference compound with high intestinal permeability documented in Caco-2 model was also analyzed. Before the experiments, the Caco-2 monolayers were washed twice with HBSS and pre-incubated at 37 °C for 30 min. Transport was initiated by adding HBSS to the acceptor (basolateral) side and the analyzed compound to the donor (apical) side, which mimicked absorptive intestinal transport. The Caco-2 cell cultures were placed in the incubator (37 °C) and continuously agitated using a plate shaker (120 rpm). At 20 min intervals, a sample was taken from the acceptor compartment, and HPLC analyzed the concentration of the transported compound. Each sample volume was replaced with fresh HBSS warmed to 37 °C. To quantify the transport of compound across the Caco-2 cell monolayer, the apparent permeability coefficient (P_app_) was determined according to the protocol previously described by Tavelin et al. [53]. Unless specified otherwise, all chemicals applied in Caco-2 cell culture and transepithelial transport experiments were purchased from Merck KGaA (Darmstadt, Germany).

### 4.4. LC-MS Analysis

The concentrations of the tested compounds in each well of the acceptor and donor plates were determined by LC-MS technique.

The amount of the compounds in question was quantified using a Shimadzu Nexera X2 UHPLC system equipped with a mass spectrometry detector (Shimadzu LCMS-2020 detector, Tokyo, Japan). The chromatography was performed on ReproSil Pure 120 ODS-3 column (Dr. Maisch GmbH, Ammerbuch-Entringen, Germany, 100 × 2 mm, 2.4 µm particle size). The column temperature was maintained at 40 °C, and for all the experiments, the flow rate was 0.3 mL/min. The mobile phase consisted of 0.1% FA + 0.05% TFA in ACN (solvent A) and 0.1% FA + 0.05% TFA in water (solvent B). Chromatographic separation was carried out using several gradient methods of solvent A for 15 min. The automatic sampler was maintained at 4 °C and the injection volume was 10 μL.

The compounds were detected and analyzed by an ESI-MS detector operated in the positive ionization mode with the use of the selected ion monitoring mode (SIM). The capillary voltage was set at 5000 V, source temperature was 300 °C, desolvation temperature was 250 °C, nebulizing gas flow rate was 1.5 L N_2_/min, and drying gas flow rate was 15 L N_2_/min. LC-MS chromatograms were recorded for selected *m*/*z* ions characteristic for the tested compounds. Chromatograms for the most abundant ions (target ions) were used for the quantification of compounds. The amount of compounds was estimated based on the integration of the peak areas on LC-MS chromatograms recorded for target ions and calibration curves prepared for the mentioned compounds.

## Figures and Tables

**Table 1:** List of synthesized compounds, their masses and ClogP values.

No	Name *^a^*	Structure	Calculated [M+H]^+^	Found [M+H]^+^	CLogP ^*b*^
1.	OT	Cys(&)-Tyr-Ile-Gln-Asn-Cys(&)-Pro-Leu-Gly-*NH*_2_	1004.444	1007.514	−0.654
2.	Et-OT	Et-Cys(&)-Tyr-Ile-Gln-Asn-Cys(&)-Pro-Leu-Gly-*NH*_2_	1079.466	1079.586	0.461
3.	MeOEt-OT	MeOEt-Cys(&)-Tyr-Ile-Gln-Asn-Cys(&)-Pro-Leu-Gly-*NH*_2_	1109.476	1109.520	0.129
4.	Prop-OT	Prop-Cys(&)-Tyr-Ile-Gln-Asn-Cys(&)-Pro-Leu-Gly-*NH*_2_	1093.481	1093.491	0.990
5.	But-OT	But-Cys(&)-Tyr-Ile-Gln-Asn-Cys(&)-Pro-Leu-Gly-*NH*_2_	1107.497	1107.541	1.519
6.	Hoc-OT	Hoc-Cys(&)-Tyr-Ile-Gln-Asn-Cys(&)-Pro-Leu-Gly-*NH*_2_	1135.528	1135.622	2.577
7.	Oct-OT	Oct-Cys(&)-Tyr-Ile-Gln-Asn-Cys(&)-Pro-Leu-Gly-*NH*_2_	1163.559	1163.635	3.635
8.	Dec-OT	Dec-Cys(&)-Tyr-Ile-Gln-Asn-Cys(&)-Pro-Leu-Gly-*NH*_2_	1191.591	1191.611	4.693
9.	Dod-OT	Dod-Cys(&)-Tyr-Ile-Gln-Asn-Cys(&)-Pro-Leu-Gly-*NH*_2_	1219.622	1219.691	5.751

*^a^* OT is oxytocin; Et–ethyloxycarbonyl group; MeOEt–2-methoxyethyloxycarbonyl; Prop–propyloxycarbonyl; But–butyloxycarbonyl; Hoc–hexyloxycarbonyl; Oct–octyloxycarbonyl; Dec–decyloxycarbonyl; Dod–dodecyloxycarbonyl. *^b^* ClogP–(calculated logP) is a computational estimate of the logP value of a compound.

**Table 2:** The permeability coefficients (P_app_) obtained in the PAMPA for OT and its prodrugs.

Compound	P_app_ (10^−6^ cm/s) ± SD	Mass Retention
Caffeine	21.99 ± 11.16	26%
Atenolol	1.24 ± 1.28	12%
OT	2.22 ± 0.21	19%
Et-OT	2.78 ± 0.17	16%
MeOEt-OT	2.37 ± 0.52	15%
Prop-OT	2.97 ± 0.64	33%
But-OT	2.20 ± 0.30	30%
Hoc-OT	4.67 ± 0.66	43%
Oct-OT	5.93 ± 0.86	33%
Dec-OT	9.28 ± 6.11	32%
Dod-OT	1.08 ± 0.95	67%

## Data Availability

All relevant data are included in the manuscript.

## Supplementary Materials

The following supporting information can be downloaded at: https://www.mdpi.com/article/10.3390/ijms26104772/s1.
