# Supplementary material for: Application of Lipophilic Prodrug Charge Masking Strategy to Obtain Novel, Potential Oxytocin Prodrugs
Source: Int J Mol Sci. 2025 May 16;26(10):4772. doi: 10.3390/ijms26104772 (PMC12112033; doi:10.3390/ijms26104772)
Supplement: Supplementary file 1 [file ijms-26-04772-s001.zip › ijms-3608945-supplementary.pdf]

Supporting Information

Application of Lipophilic Prodrug Charge Masking strategy to obtain novel, potential oxytocin prodrugs

Agata Gitlin-Domagalska[1*], Anna Olejnik[2], Jarosław Ruczyński[1*], Dominika Starego[1], Natalia Ptaszyńska[1], Anna Łęgowska[1], Dawid Debowski [1], Chaim Gilon[3], Krzysztof Rolka[1]

[1] Department of Molecular Biochemistry, Faculty of Chemistry, University of Gdansk, Gdansk, Poland

[2] Department of Biotechnology and Food Microbiology, Faculty of Food Science and Nutrition, Poznan University of Life Sciences, Poznań, Poland

[3] Institute of Chemistry, The Hebrew University of Jerusalem, Jerusalem, Israel

Table of contents

Figure S1. MS analysis of OT; 3
Figure S2. MS analysis of Et-OT; 4
Figure S3. MS analysis of MeOEt-OT; 5
Figure S4. MS analysis of Prop-OT; 6
Figure S5. MS analysis of But-OT; 6
Figure S6. MS analysis of Hoc-OT; 7
Figure S7. MS analysis of Oct-OT;...8
Figure S8. MS analysis of Dec-OT; 9
Figure S9. MS analysis of Dod-OT; 10
Figure S10. HPLC analysis of OT...11
Figure S11. HPLC analysis of Et-OT. 11
Figure S12. HPLC analysis of MeOEt- OT. 12
Figure S13. HPLC analysis of Prop-OT. 12
Figure S14. HPLC analysis of But-OT. 13
Figure S15. HPLC analysis of Hoc-OT. 13
Figure S16. HPLC analysis of Oct-OT..14
Figure S17. HPLC analysis of Dec-OT 14
Figure S18. HPLC analysis of Dod-OT. 15
Figure S19. Transepithelial electrical resistance (TEER) during long-term Caco-2 cell culture to form integrated intestinal epithelium. 15

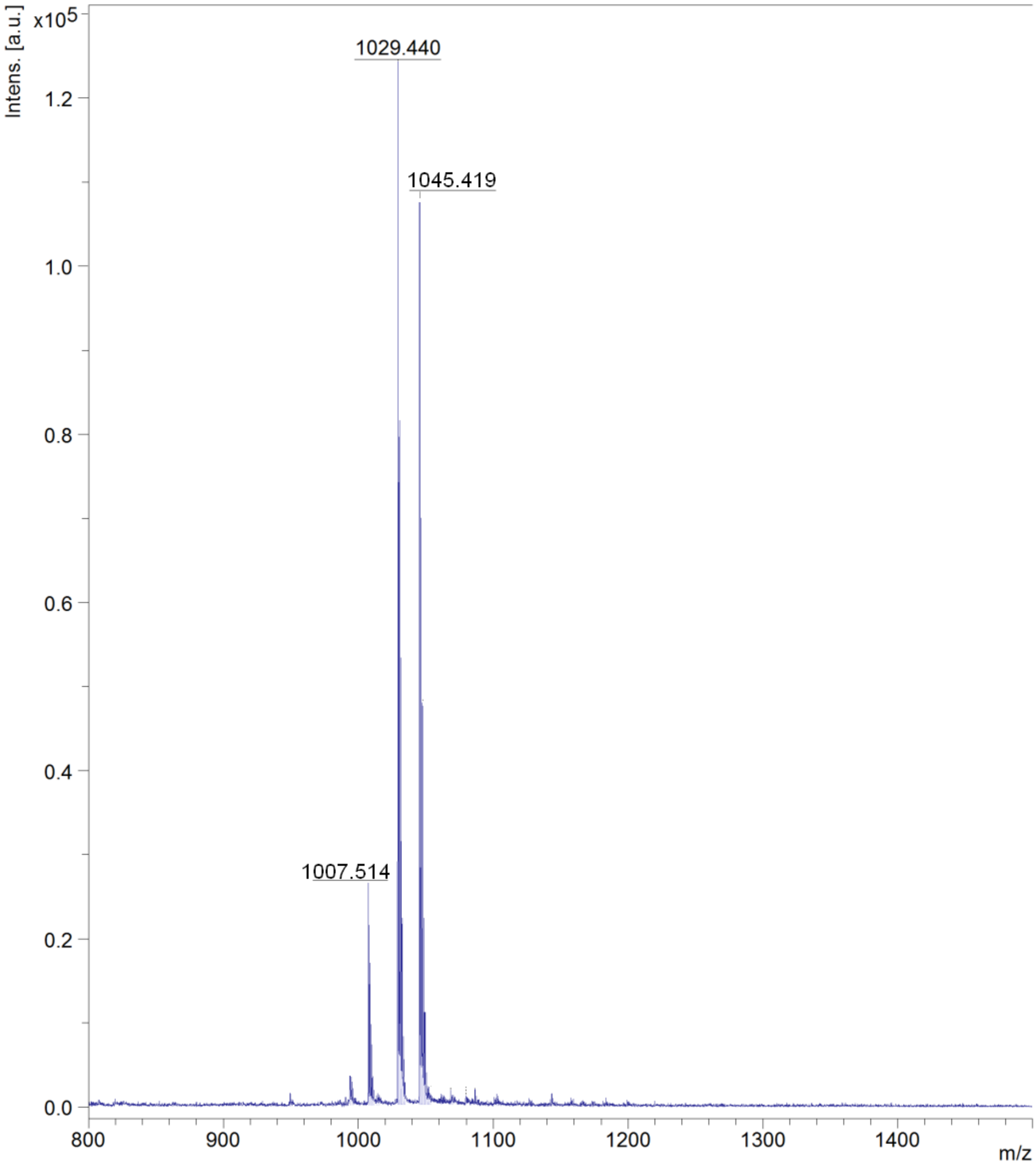

Figure S1. MS analysis of OT; Calculated mass 1006.44; Found mass $[M+H]^+$ 1007.514, $[M+Na]^+$ 1029.440, $[M+K]^+$ 1045.419

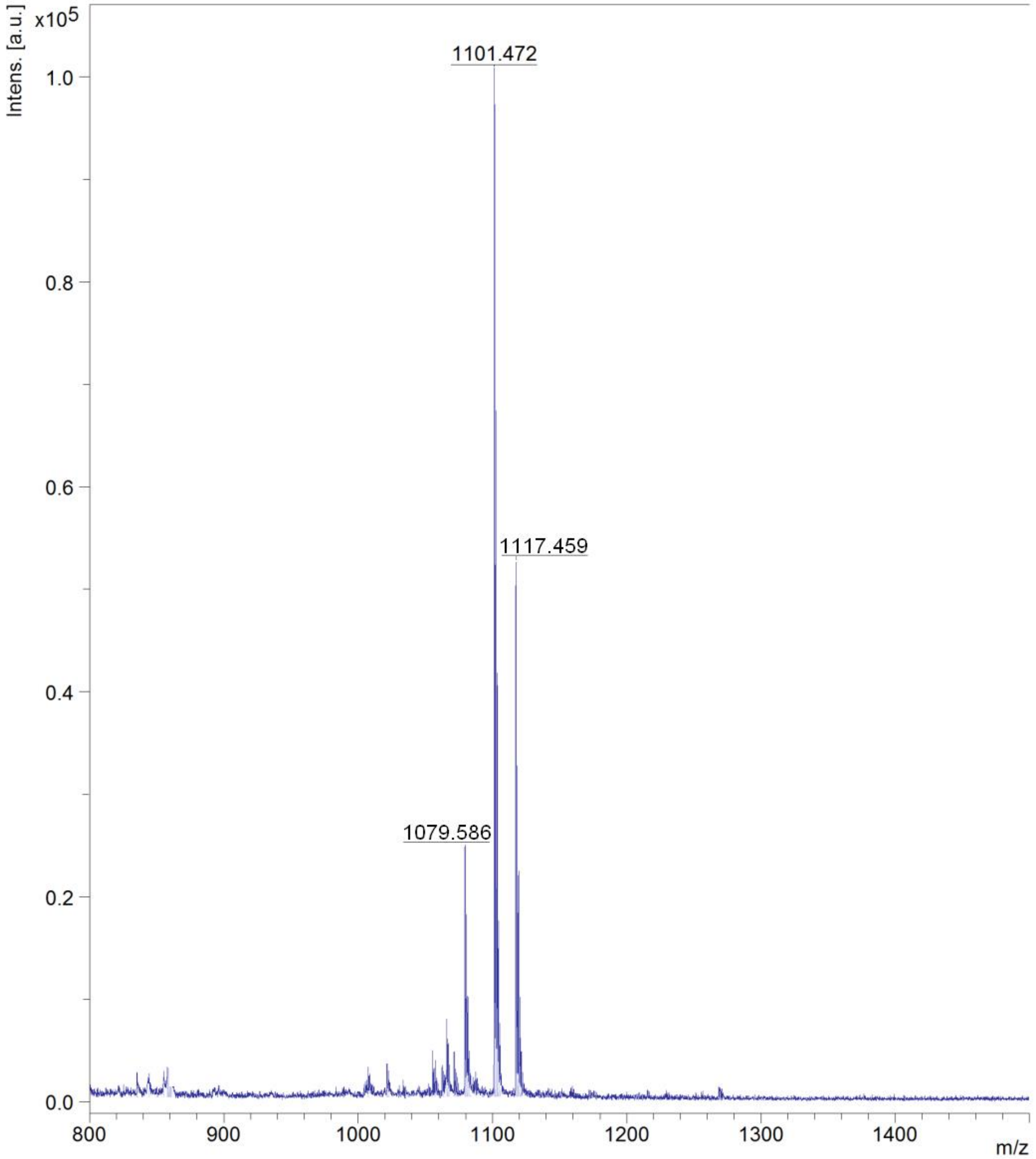

Figure S2. MS analysis of Et-OT; Calculated mass 1078.46; Found mass $[M+H]^+$ 1079.586 $[M+Na]^+$ 1101.472, $[M+K]^+$ 1117.459

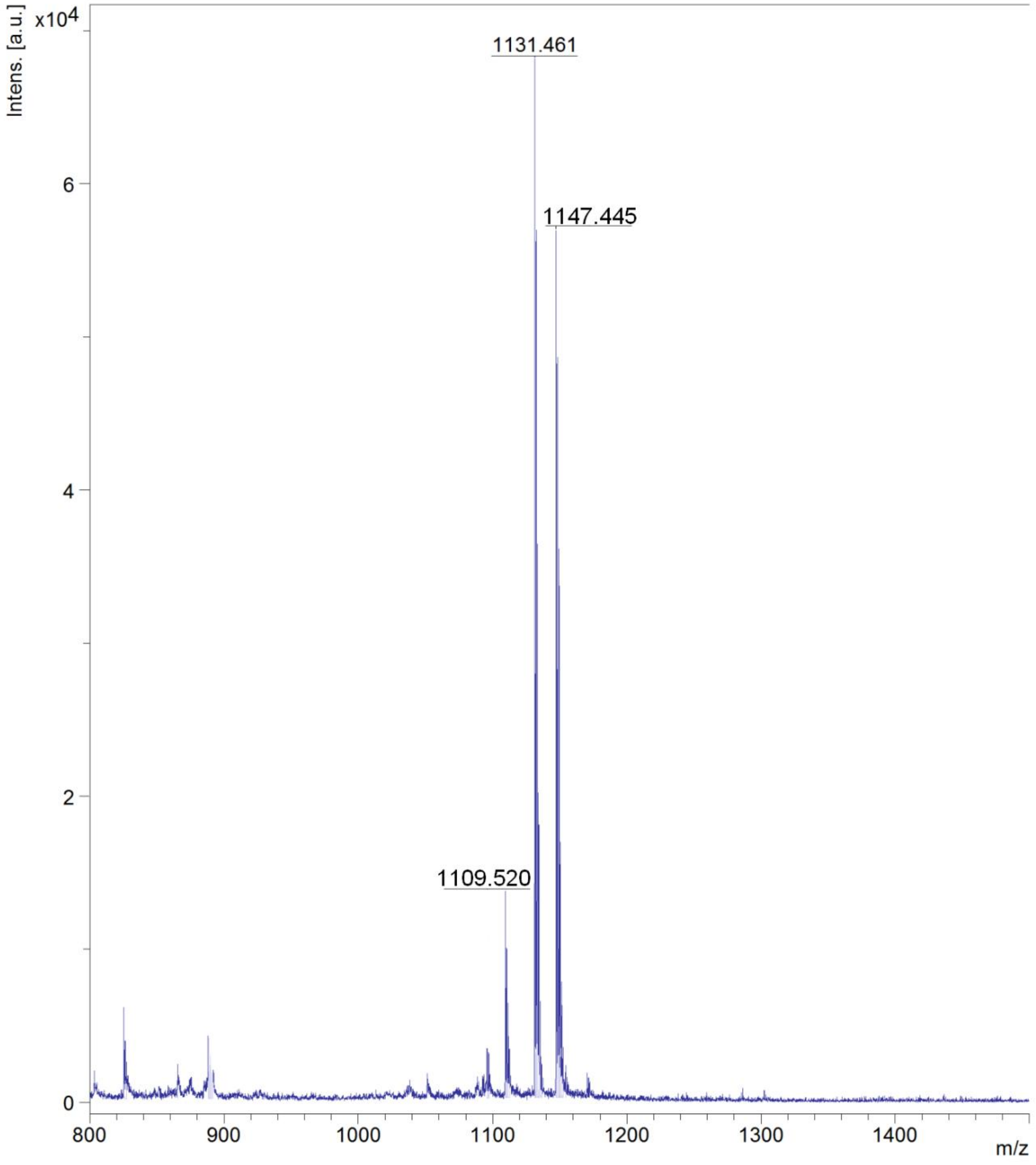

Figure S3. MS analysis of MeOEt-OT; Calculated mass 1108.47; Found mass $[M+H]^+$ 1109.520, $[M+Na]^+$ 1131.461, $[M+K]^+$ 1147.445

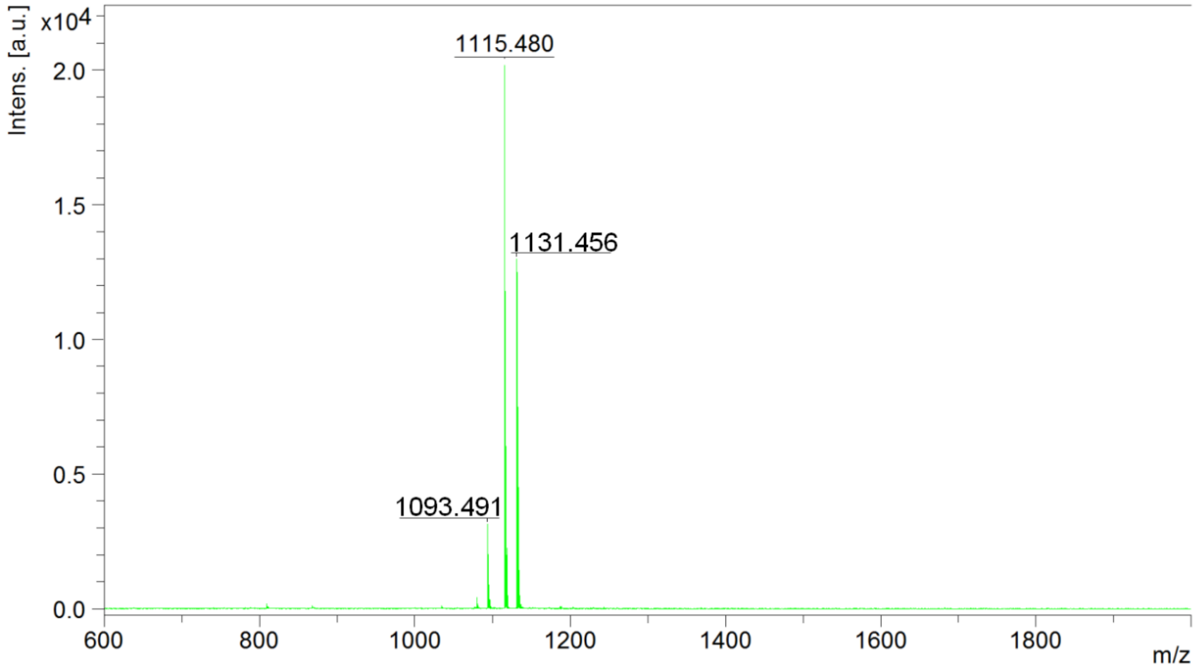

Figure S4. MS analysis of Prop-OT; Calculated mass 1092.47; Found mass $[M+H]^+$ 1093.491, $[M+Na]^+$ 1115.480, $[M+K]^+$ 1131.456

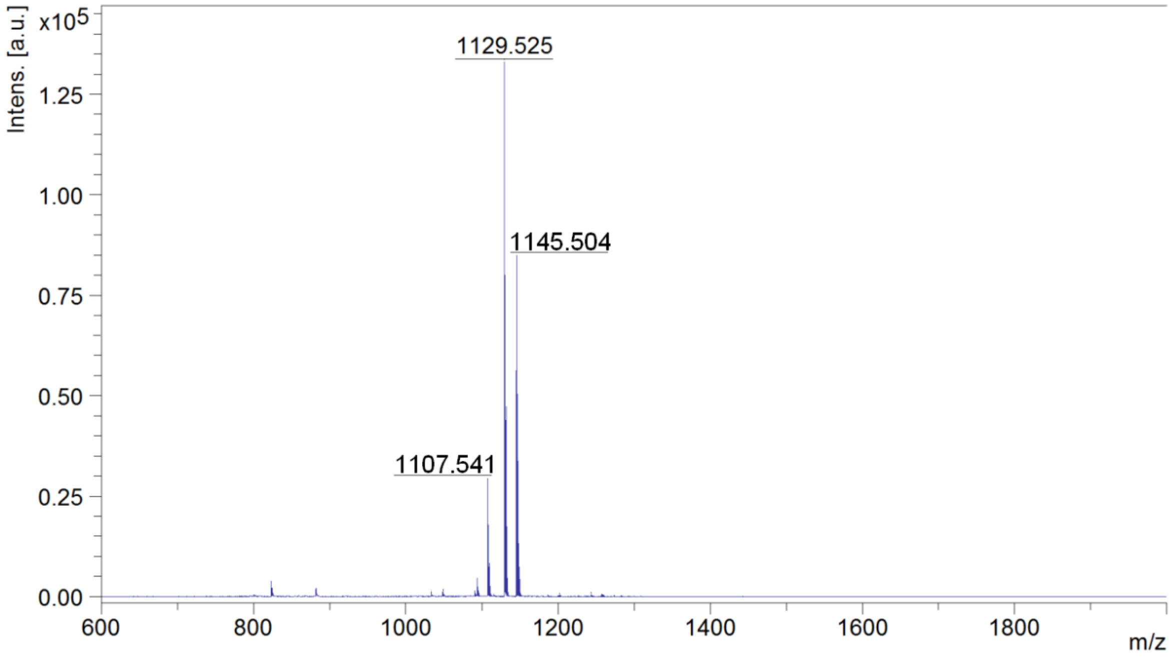

Figure S5. MS analysis of But-OT; Calculated mass 1106.49; Found mass $[M+H]^+$ 1107.541, $[M+Na]^+$ 1129.525, $[M+K]^+$ 1145.504

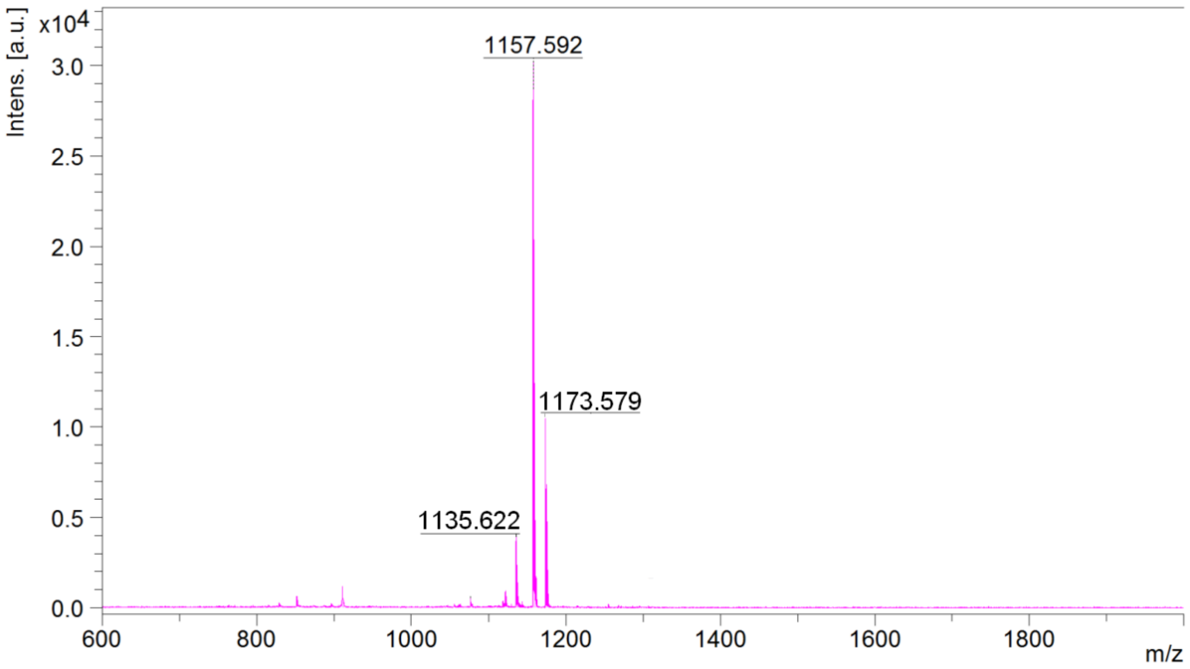

Figure S6. MS analysis of Hoc-OT; Calculated mass 1134.52; Found mass [M+H]$^+$ 1135.622, [M+Na]$^+$ 1157.582, [M+K]$^+$ 1173.579

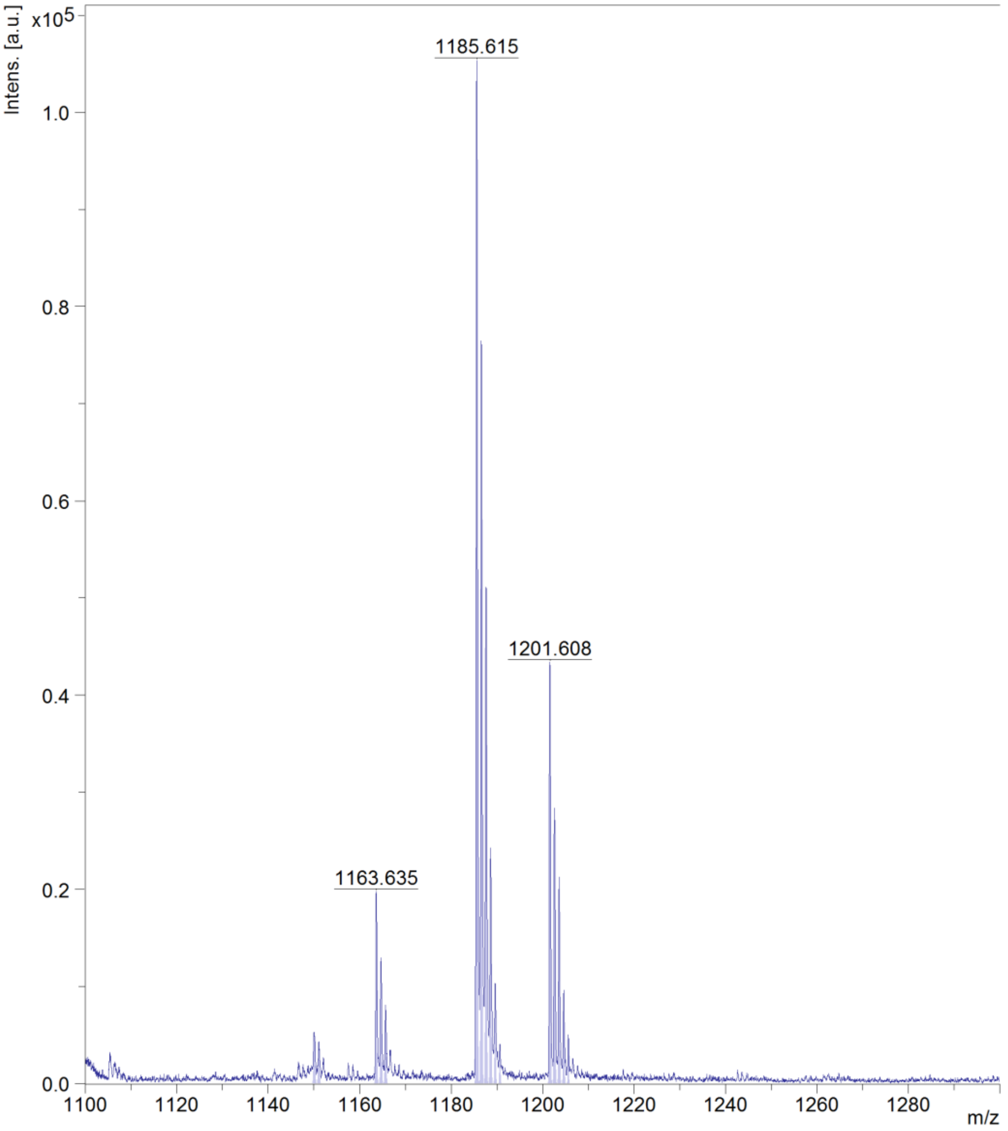

Figure S7. MS analysis of Oct-OT; Calculated mass 1162.55; Found mass $[M+H]^+$ 1163.635, $[M+Na]^+$ 1185.615, $[M+K]^+$ 1201.608

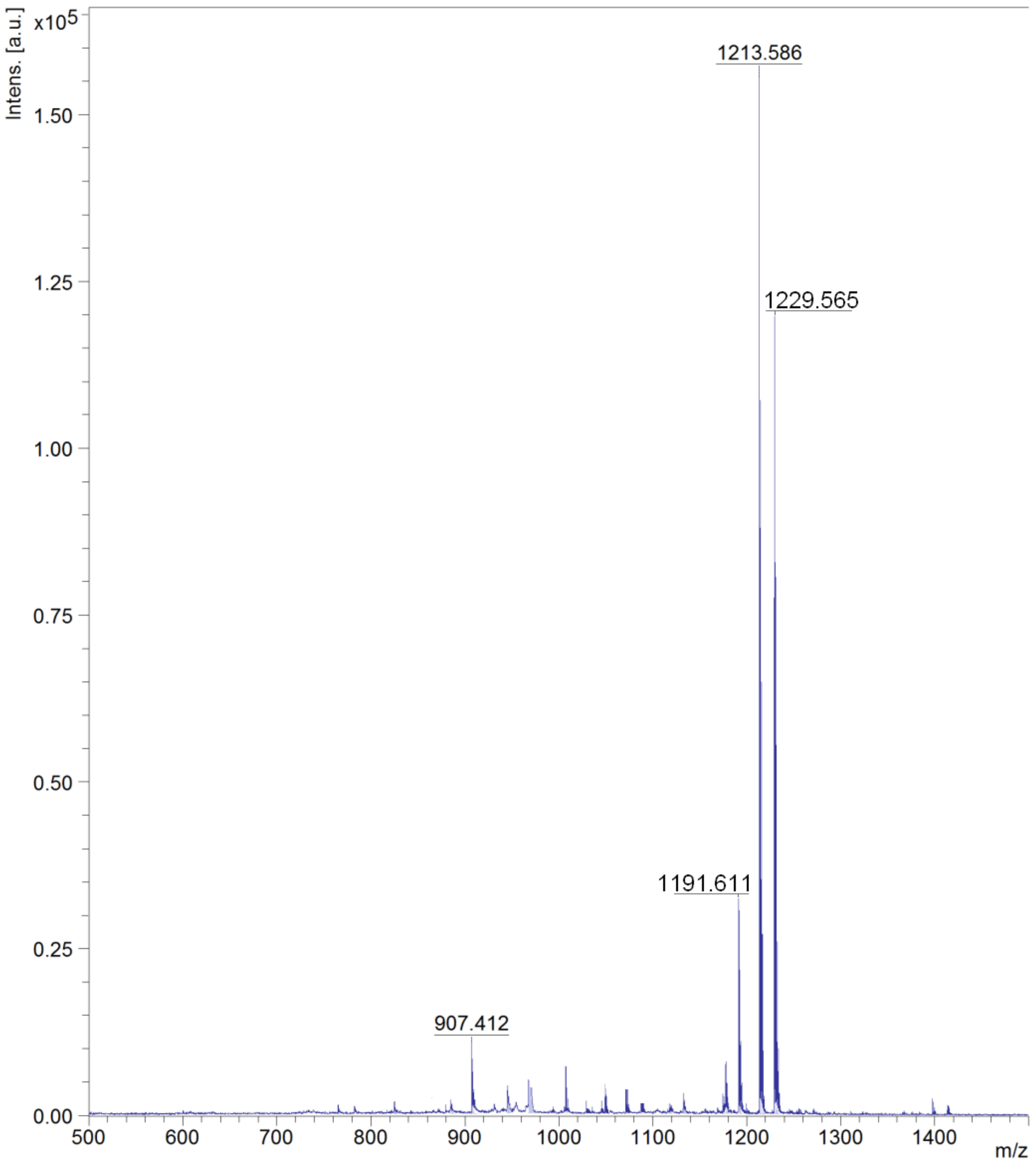

Figure S8. MS analysis of Dec-OT; Calculated mass 1190.58; Found mass $[M+H]^+$ 1191.611, $[M+Na]^+$ 1213.586, $[M+K]^+$ 1229.565

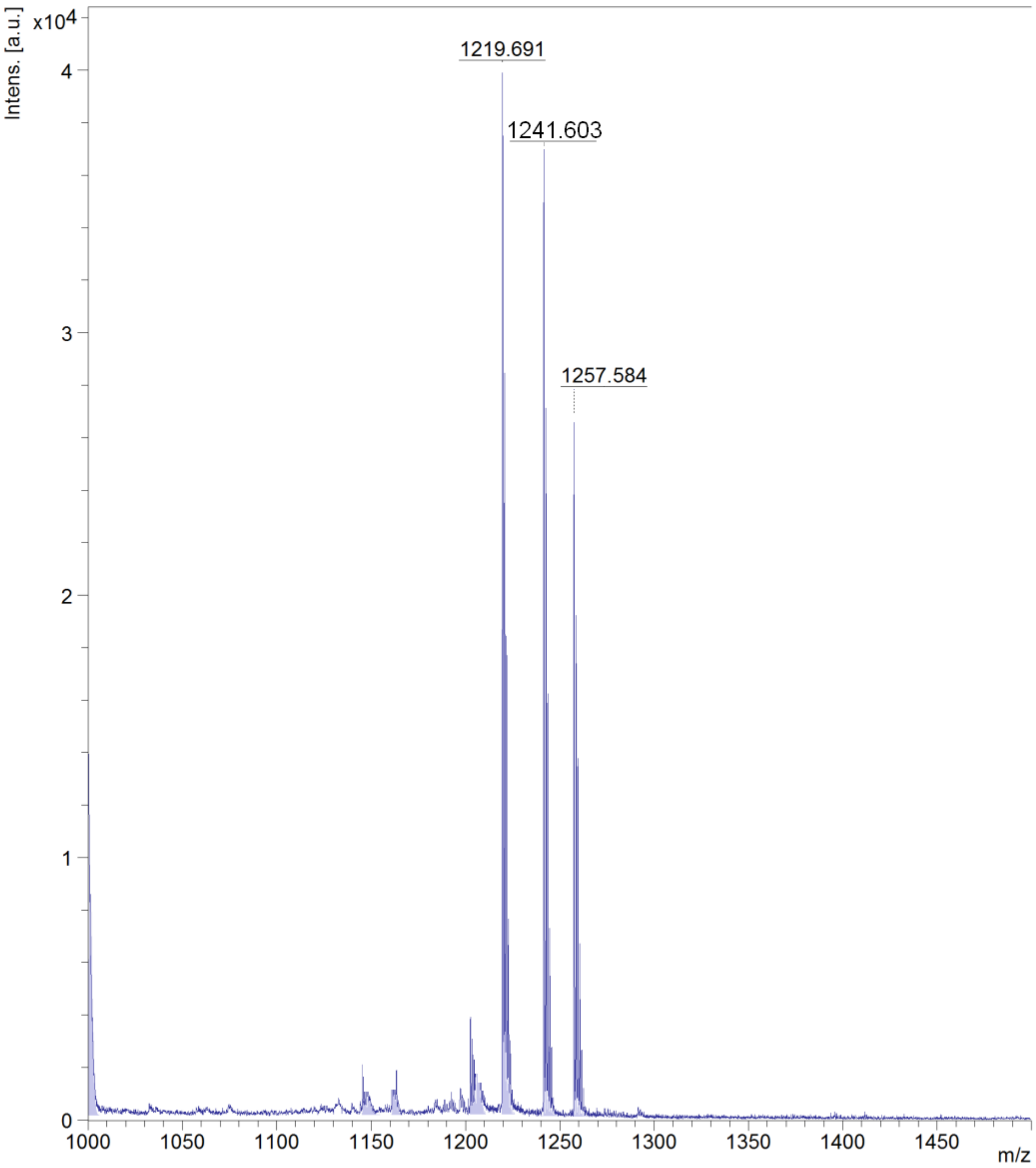

Figure S9. MS analysis of Dod-OT; Calculated mass 1218.61; Found mass $[M+H]^+$ 1219.691, $[M+Na]^+$ 1241.603, $[M+K]^+$ 1257.584

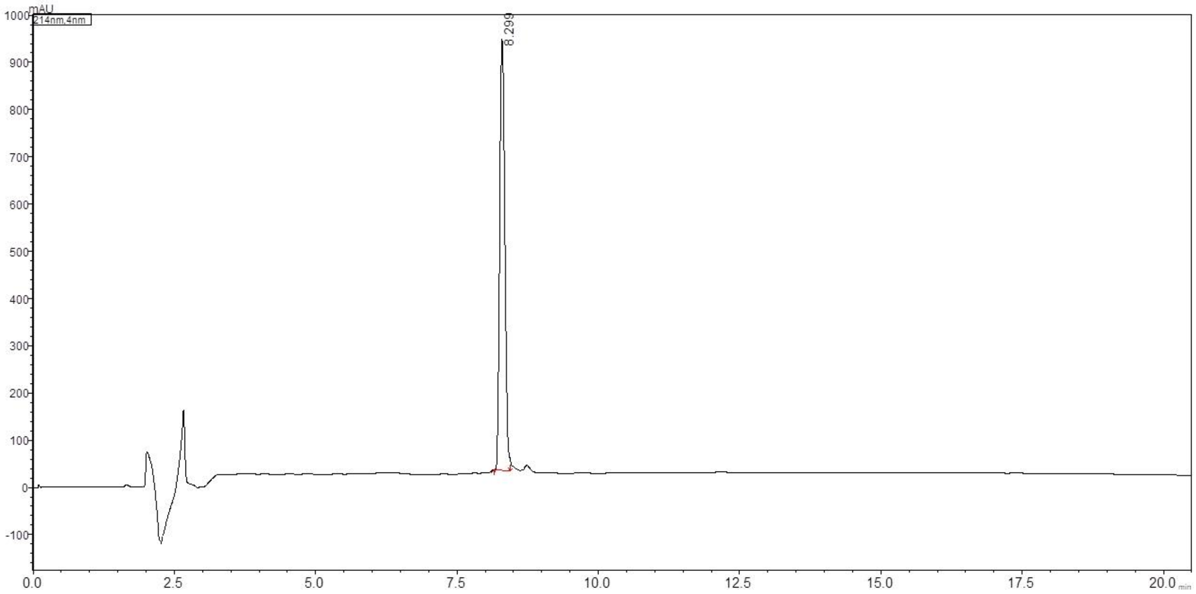

Figure S10. HPLC analysis of OT.

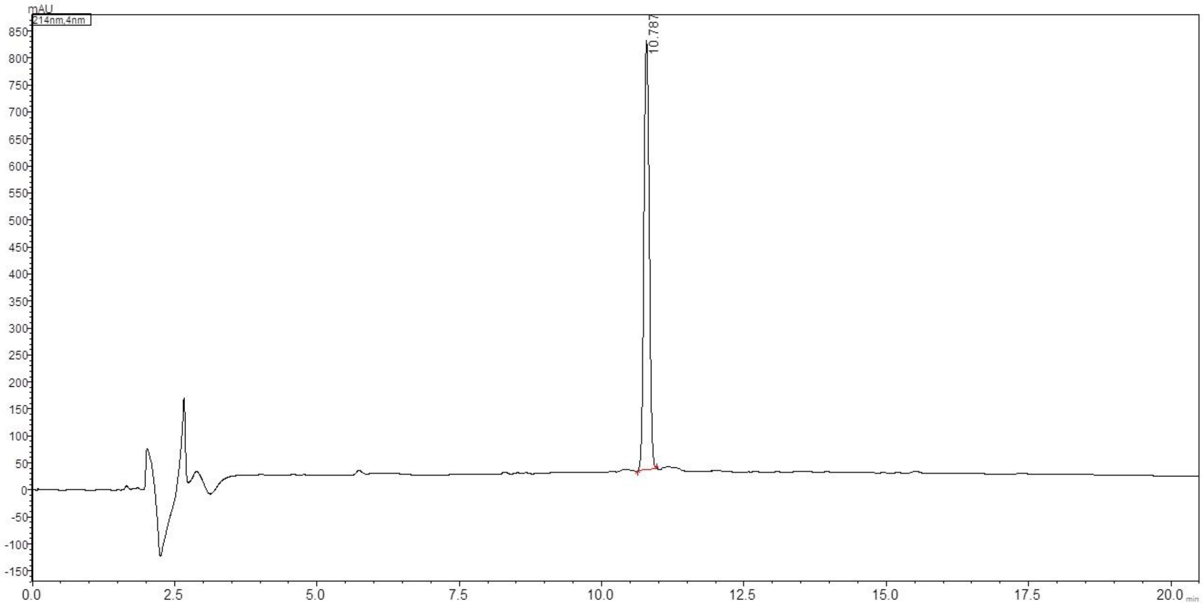

Figure S11. HPLC analysis of Et-OT.

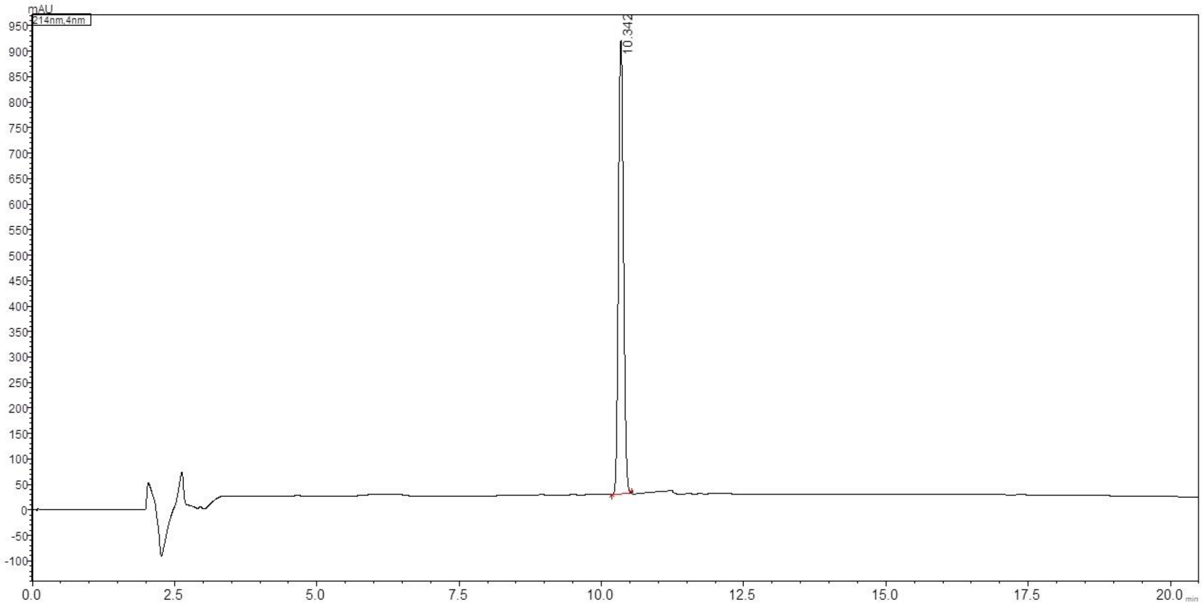

Figure S12. HPLC analysis of MeOEt- OT.

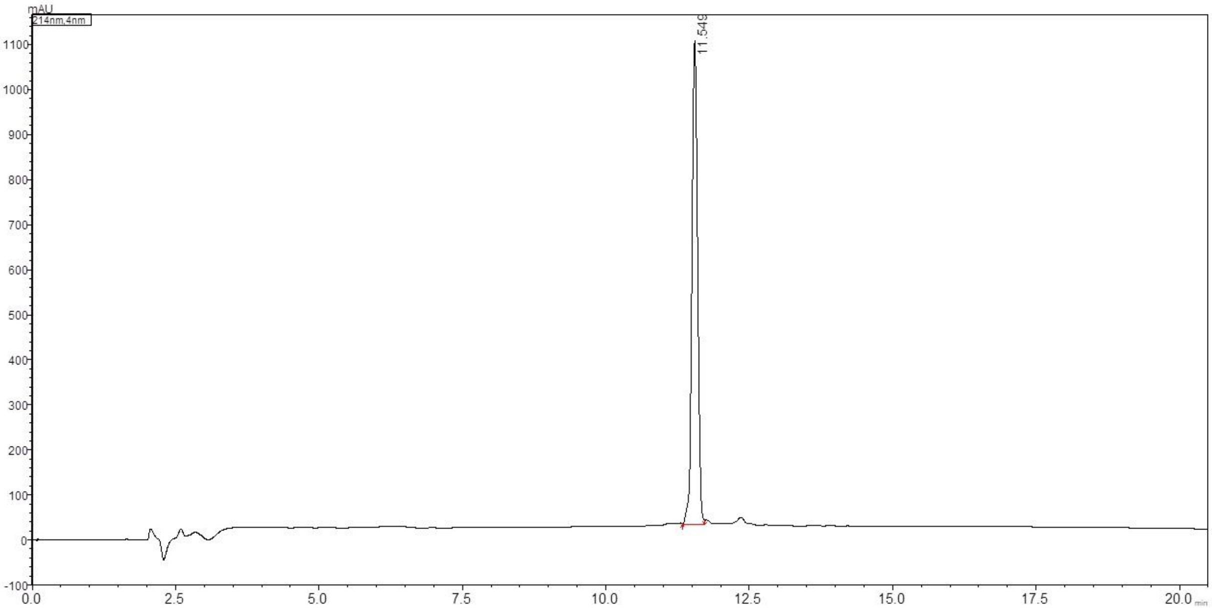

Figure S13. HPLC analysis of Prop-OT.

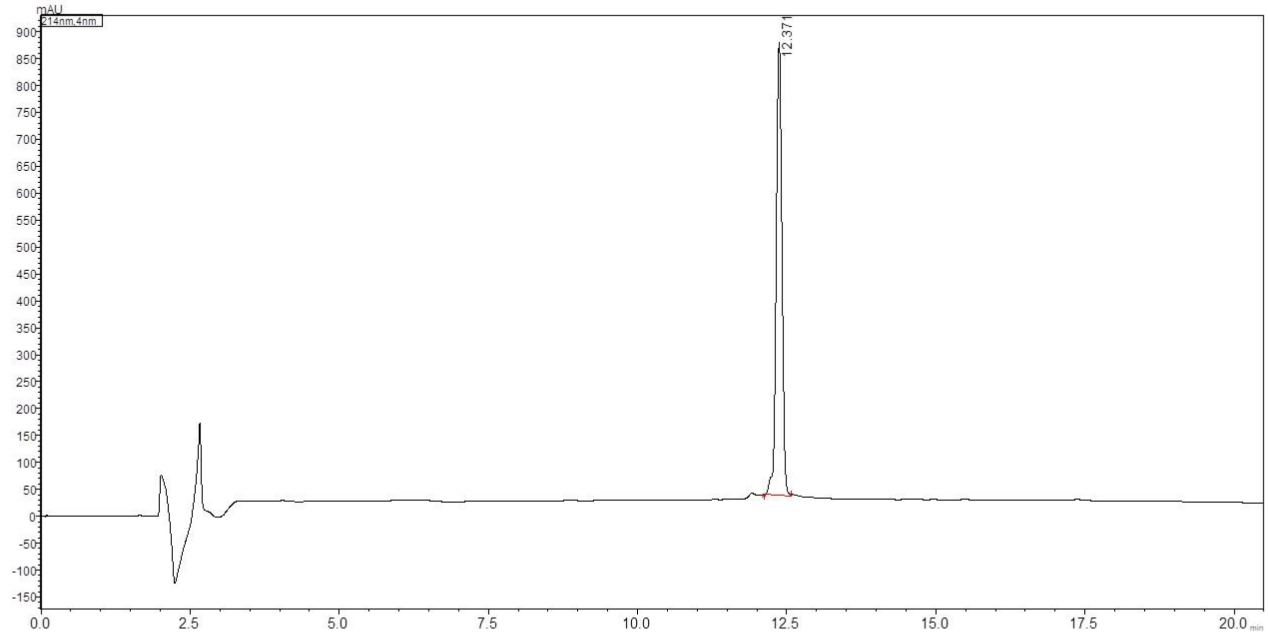

Figure S14. HPLC analysis of But-OT.

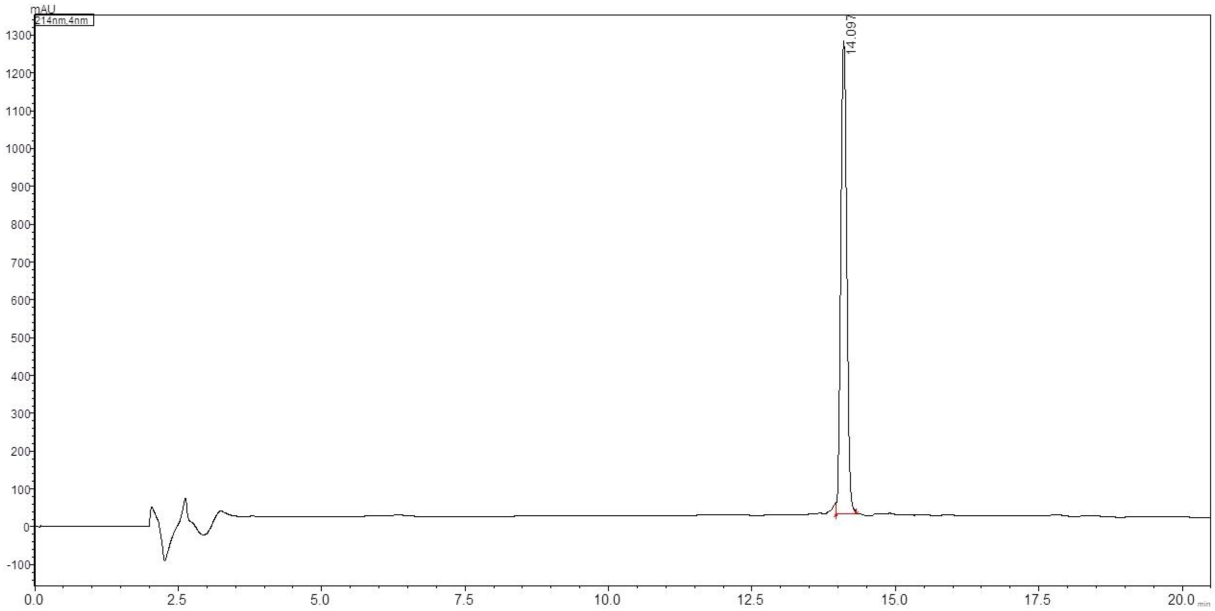

Figure S15. HPLC analysis of Hoc-OT.

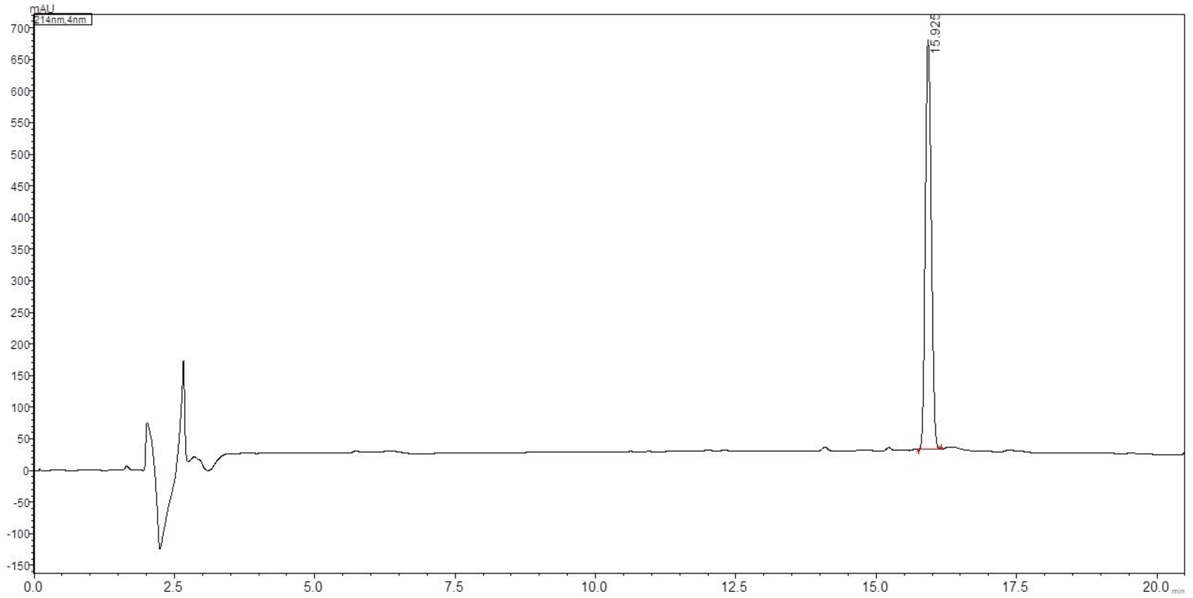

Figure S16. HPLC analysis of Oct-OT

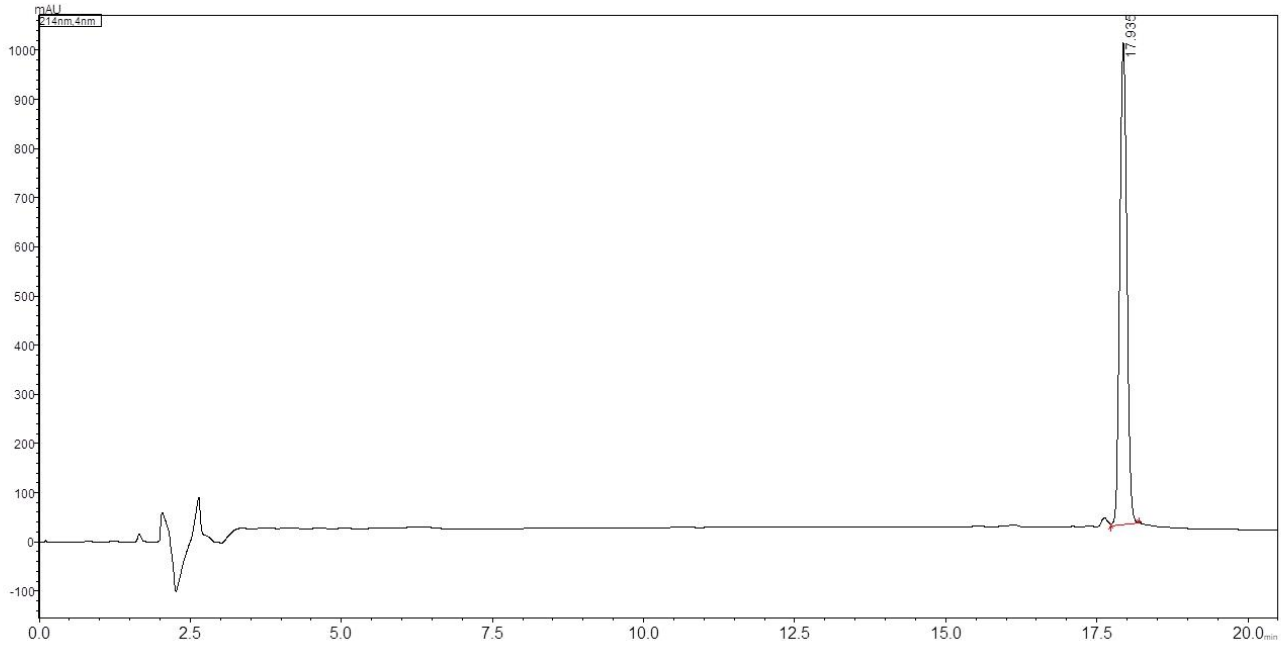

Figure S17. HPLC analysis of Dec-OT

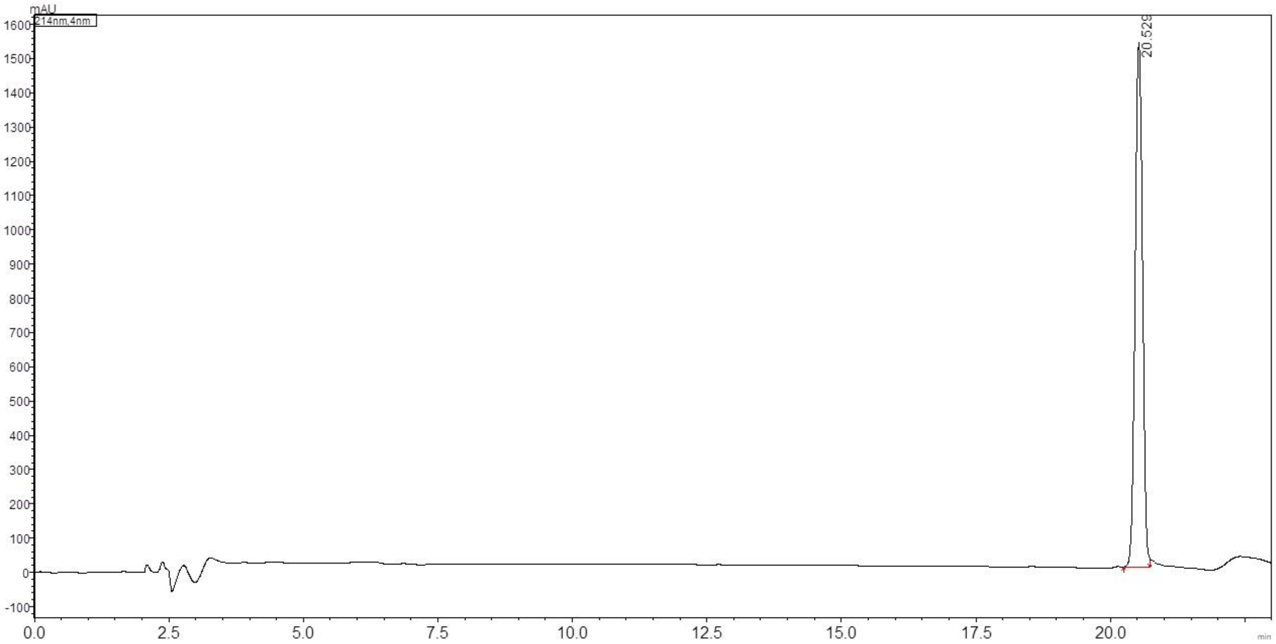

Figure S18. HPLC analysis of Dod-OT. This compound was analyzed in standard method (10-90%B in 20 min), however the time of chromatogram view was extended to 23 min.

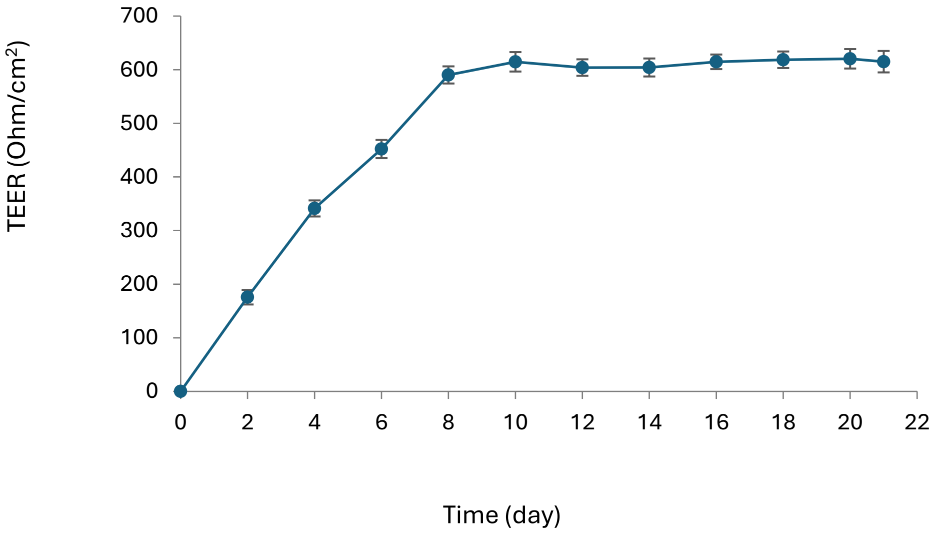

Figure S19. Transepithelial electrical resistance (TEER) during long-term Caco-2 cell culture to form integrated intestinal epithelium. Data present mean TEER values (±SD) obtained from 10 Caco-2 cell cultures.
